# MicroRNA as key players in skin regeneration and anti-aging pathways

**DOI:** 10.15698/cst2026.10.322

**Published:** 2026-10-12

**Authors:** Hanieh Azizi, Reza Moghadasali, Amir Mohammad Amirkhani, Ehsan Taghiabadi, Mohammad Ali Nilforoushzadeh

**Affiliations:** 1 Department of Advanced Biological Sciences and Technologies, University of Science and Culture, Tehran, Iran; 2 Department of Stem Cells and Developmental Biology, Cell Science Research Center, Royan Institute for Stem Cell Biology and Technology, ACECR, Tehran, Iran; 3 Skin and Stem Cell Research Center, Tehran University of Medical Science, Iran; 4 Skin Repair Research Center, Jordan Dermatology and Hair Transplantation Center, Tehran, Iran

**Keywords:** microRNA, anti-aging therapy, skin rejuvenation, inflammation regulation

## Abstract

MicroRNAs (miRNAs) are a class of small non-coding RNAs that significantly impact skin rejuvenation via gene regulation that is involved in mechanisms such as regeneration, wound repair, and aging. By potentially shifting pathways modulating inflammation, cell proliferation, and extracellular matrix remodeling, they can be considered as an effective anti-aging intervention candidate. Such molecular mechanisms of miRNAs in cutaneous homeostasis of fibroblast activity, keratinocyte differentiation, and collagen synthesis are provided in the current review. Representative miRNAs such as miRNA-124, miRNA-377, miRNA-34 family, and miRNA-29 family, miRNA-302b-3p, miRNA-361-5p, miRNA-146a, miRNA-21, miRNA-181-a, miRNA155 demonstrate distinct roles in regulating wound healing, inflammatory resolution, maintaining the extracellular matrix, and handling oxidative stress. This review highlights current knowledge of miRNA-mediated regulation in skin regeneration and aging and synthesizes recent findings on their individual functions in these biological processes. It also emphasizes the potential of miRNA-based interventions as a next generation approach to skin rejuvenation, while addressing existing challenges that must be resolved before these strategies can be translated into clinical applications.

## INTRODUCTION

The skin comprises three layers: the epidermis, the dermis, and the hypodermis. The outermost layer, known as the epidermis, protects the body and contains cells like keratinocytes and melanocytes that secrete pigment and protect it from UV damage. Underneath, the dermis offers both strength and elasticity with the presence of collagen fibers, supporting the vascular system, nerve endings, glands, and hair follicles that serve sensation and renewal. The deepest layer is the hypodermis, composed mostly of fat cells to cushion muscles and bones but to insulate the body and regulate temperature 1.

Skin aging results from the interplay of intrinsic biological processes and extrinsic environmental exposures. Intrinsic aging reflects genetically programmed and metabolic changes that progressively reduce cellular function, whereas extrinsic factors most notably ultraviolet radiation, pollution, and lifestyle-associated stressors accelerate structural and biochemical deterioration. Together, these influences lead to thinning of the epidermis, impaired barrier integrity, and extensive remodeling of the dermal extracellular matrix (ECM) 2.

More broadly, aging is a complex, multifactorial process characterized by progressive structural and functional decline across cells, tissues, and organs 3. The skin provides one of the most apparent reflections of this trajectory, as intrinsic physiological changes combined with cumulative environmental damage to produce impaired barrier function, metabolic dysregulation, diminished repair mechanisms, and degradation of structural proteins. Collectively, these processes underlie the clinical and histological features associated with aged skin 4.

A key driver of these changes is the age-related decline in fibroblast function. Fibroblasts are the predominant cells in dermal tissues and produce collagen and other ECM constituents which maintain the structural integrity of the skin. Fibroblast changes under aging, such as cell cycle dysregulation, DNA damage, apoptosis, and impaired ECM turnover, are related to dermal homeostasis and function. Fibroblast senescence is thus identified as a key driver of cutaneous aging. Excessive oxidative stress, genetic and epigenetic alterations, mitochondrial dysfunction, and age-associated decline in immune surveillance are some of the mechanisms that drive this process 5.

Epigenetic changes, such as DNA methylation, histone changes, and chromatin remodeling, are also believed to play a crucial role in aging processes 6. The most important task of DNA methyltransferase 1 (DNMT1), which Bestor 7 revealed for the first time in 1988, is to maintain DNA methylation after replication and promote transfer of this methylation state to descendant cells. Global levels and patterns of DNA methylation with skin aging were detected in a high-throughput study. In one study 8, Tet2, DNMT3a, and DNMT3b expression significantly reduced in mouse skin as the skin aged, but few previously investigated DNA methyltransferase roles and mechanisms for skin aging. Repeated and unprocessed cellular stress can lead to cellular senescence. MiRNA response is one of the chemical reactions that cells undergo to stress 9. MiRNAs refer to small, naturally occurring, single-stranded RNA that does not code for proteins. To accomplish this, they interact with a portion of a target mRNA molecule known as the 3’ UTR. At this point, the translation process either stops, or the mRNA molecule is fragmented, enabling the operation of regulating the expression of several genes 10. Studies have associated miRNA profiles with age-related properties, supporting the underlying role of miRNA profiles in multifaceted cellular functions and systemic aging processes overall 11.

## BIOGENESIS OF MicroRNAs

As of the present date, about 2700 human mature miRNAs have been identified, and they are responsible for targeting over 60% of human protein-coding genes. In addition, miRNAs are believed to regulate the expression of almost one-third of all human genes 12. miRNAs are a class of small, non-coding, endogenous, evolutionarily conserved RNAs that are roughly 19–24 nucleotides long. Their main functions are to control translation and messenger RNA (mRNA) levels. Their primary function is post-transcriptional regulation of gene expression 13.

The ability of miRNAs to cleave mRNA targets and prevent translation results in decreased protein levels. Additionally, miRNA binding has been demonstrated to activate mRNA decay factors, increasing mRNA instability and degradation and ultimately lowering expression levels 14. Moreover, a variety of pathobiological processes, such as cancer, age-related illnesses, tissue degradation, apoptosis/necrosis, differentiation, and cell proliferation, have been shown to be influenced by miRNAs 15.

The biogenesis of miRNAs occurs through a multi-step process that requires both nuclear and cytoplasmic phases. In the nucleus, RNA polymerase II and III normally transcribe miRNAs as a long primary miRNA (pri-miRNA) with a cap and a poly-A tail. Pri-miRNA has a terminal loop composed of a double-stranded stem of about 30 base pairs and two flanking unstructured single-stranded tails. The RNase III enzyme Drosha and the double-stranded RNA binding protein DGCR8 (Di George syndrome critical region 8 gene) make up the microprocessor complex, which breaks down pri-miRNAs into short 60–70 nucleotide stem-loop structures known as precursor miRNAs (pre-miRNAs) 16. The Exportin5 (XPO5) complex, a part of the karyopherin β family attached to the Ras-related nuclear protein (Ran) GTPase, facilitates the translocation of pre-miRNA into cytoplasm across the nuclear pore complex. Pre-miRNA discharge occurs in the cytoplasm when guanosine triphosphate (GTP) is hydrolyzed to guanosine diphosphate (GDP) 17.

Pre-miRNA is processed by the RNase III enzyme Dicer, which produces the miRNA duplex with phosphate at the 5 ${}^{\prime }$ end and a two-nucleotide overhang with a hydroxyl group at the 3 ${}^{\prime }$ end. When the RNA-induced silencing complex (RISC) is present, the miRNA duplex is loaded onto the Argonaute (AGO) protein. AGO and its companion protein, Dicer, also referred to as the transactivation response RNA binding protein (TRBP), make up RISC. The guide and passenger strands in the miRNA duplex are identified by TRBP. The protein loads the miRNA duplex onto the AGO protein in the proper orientation after identifying the strand with the less stable 5’ end. Only one strand remains to form an effector complex RISC after AGO unwinds the duplex and extracts the passenger strand while keeping the mature miRNA molecule. After its loading into RISC, the miRNA base-pairs with the target mRNA to direct the posttranscriptional repression 18. Through their interaction with the target mRNA’s 3 ${}^{\prime }$ untranslated region (UTR), miRNAs appear to have two methods of inactivating the mRNA: either by triggering mRNA degradation or by inhibiting the translation of the target mRNA. The first mechanism is based on complementary binding between miRNA and mRNA, followed by the target mRNA being deadened and degraded. The second mechanism, in which the RISC complex blocks the ribosomal subunit from binding, results in translational repression 19.

## SKIN MORPHOGENESIS AND THE ROLE OF MicroRNAs

Increasing evidence establishes miRNAs as critical epigenetic orchestrators of skin homeostasis, fine-tuning the balance between stem cell self-renewal and lineage-specific differentiation 20. For example, specific subsets of miRNAs, such as miR-125b, are preferentially expressed in skin stem cells to maintain their self-renewal capacity 21. In contrast, other miRNAs like miR-203 function as molecular switches that enforce differentiation by suppressing proliferative pathways, thereby driving the transition from basal stem/progenitor cells to stratified epithelial layers. This miRNA-mediated regulation underscores the complexity of the molecular networks governing epidermal maintenance and repair 22.

The generation of transient amplifying cells from basal epidermal stem cells is crucial for the continuous renewal of the human epidermis. These stem cells proliferate transient amplifying cells, which subsequently migrate outward through the epidermis after reaching terminal differentiation. Positioned near the basal membrane, this process involves numerous biological activities that are tightly regulated, with miRNAs potentially being a contributing factor 22. Research indicates that miRNAs are vital to this mechanism; for instance, a reduction in epithelial-specific dicer or *dgcr8* genes results in abnormal hair follicle formation, improper barrier development, and excessive proliferation of basal interfollicular keratinocytes. The most prevalently expressed miRNAs in skin include miR-152, miR-143, miR-126, miR-21, miR-27a, miR-214, miR-16, miR-203, miR-125b, miR-34a, miR-205, miR-125a, and miR-191. Additionally, there are families such as the miR-200 family (comprising miR-200a, miR-200b, miR-200c, miR-141, and miR-429), the miR-199 family (which includes both variants of mir199), as well as members from the mir19/20 family (encompassing mir19b and mir20 alongside mir17–5p and mir93) Figure 1. Moreover, it has recently been shown that in skin fibroblasts; families such as mir34 and mir29 along with mir302b–3p are associated with skin aging processes 23.

**Figure 1:**
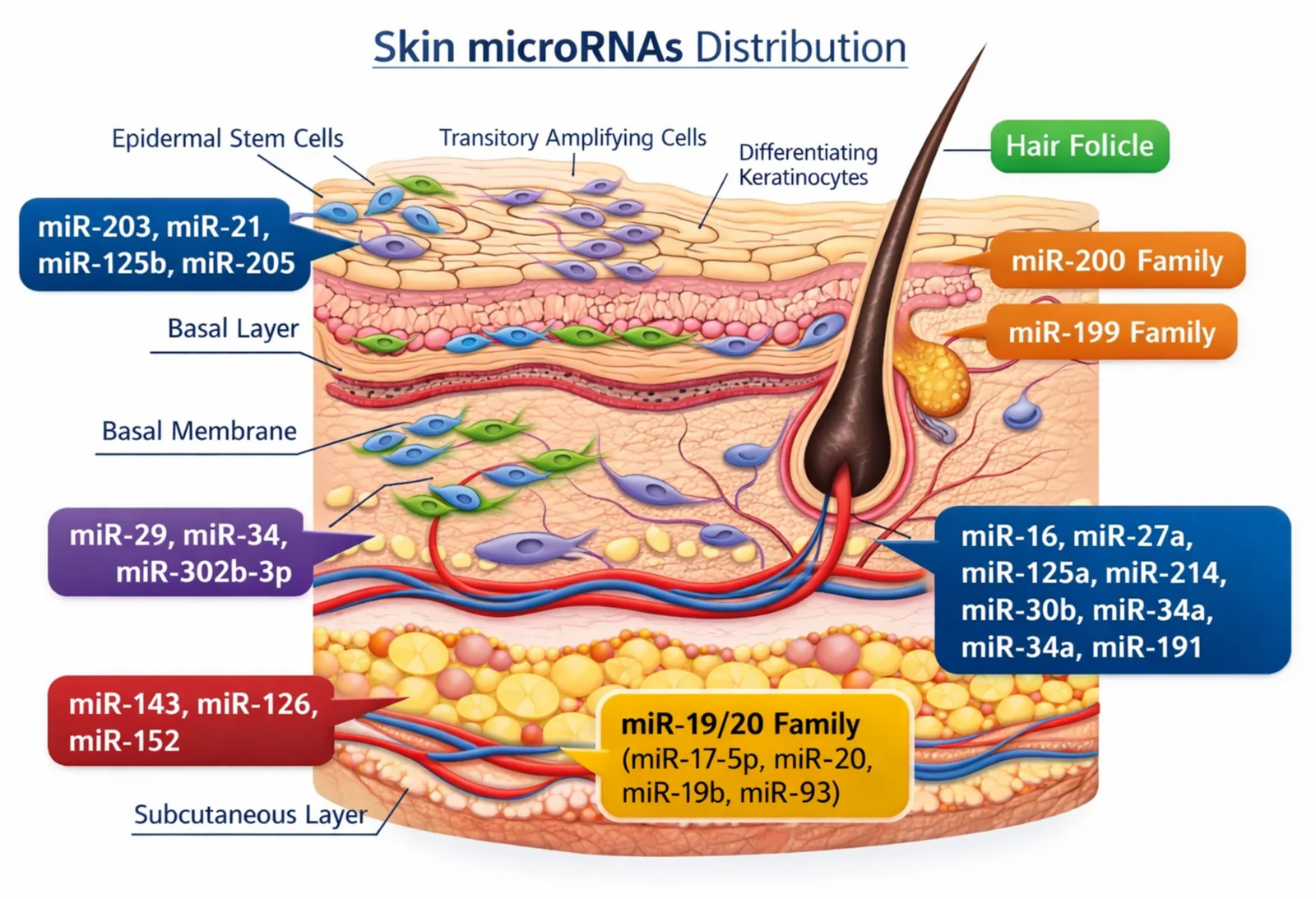
Distribution and functions of miRNAs in different layers of human skin.

## MicroRNAs’ FUNCTION IN SKIN AGING

MiRNAs and their involvement in skin control and homeostasis have become evident in recent years. Multiple evidence-based studies have elucidated the role of new miRNAs in regulating gene expression in stem cell and developmental biology. As of now, a great deal is known regarding the function of miRNAs in several stages of physiology, pathophysiology, as well as organismal development of various illnesses. Because it is unclear how miRNAs influence the skin during development, maturation, function, and aging, studies have been conducted to understand the relevant mechanisms. This is the focus for this section on the mechanisms by which miRNAs maintain the molecular regulation of the aging process and their primary targets in the skin (keratinocytes, dermal fibroblasts, melanocytes) 23.

## MicroRNAs AS REGULATORS OF KERATINOCYTE DETERIORATION

One of the first investigations profiling senescence-associated miRNAs in normal human keratinocytes (NHKs) identified 126 miRNAs that were significantly altered (fold-change > 2). Of these, a global up-regulation was observed, with 117 miRNAs (93%) showing increased expression, while only 9 miRNAs (7%) were down-regulated compared to exponentially replicating NHKs. Among all three NHK cultures, both miR-137 and miR-668 were significantly up-regulated during both organismal aging and replicative senescence. Because they also observed down-regulated levels of miR-137 and miR-668 in a variety of human head and neck squamous cell carcinoma cell lines, the researchers thought that senescence could be a potent tumor-suppressive mechanism. Moreover, they detected a direct correlation between those above-mentioned miRNAs and increments in senescence-associated β -galactosidase activity, and the senescence markers *p16INK4A* and *p53* of the ARF/p53 and p16INK4A/RB pathways, respectively. Apart from preventing cancer cells from proliferating, miR-137 is also associated with generation of cell cycle G1 arrest by targeting CDK6 and Cdc42. MiR-137 probably suppresses the Cdc42-PAK1-MLC or ERK signaling pathway and the growth/invasion of colorectal cancer cells by mimicking the impact of Cdc42 knockdown 24. miR-138, miR-181a, miR-181b and miR-130b in keratinocytes are up-regulated in replicative senescence. These four miRNAs were found to influence the pathways that regulate cell proliferation by concentrating on *p63* and *SIRT1 (Sirtuin 1)* mRNAs. They also reduced cell death produced by *p53* and *FOXO.* In addition, the NAD-dependent *SIRT1* enzyme also transformed cell differentiation, metabolism, immune response, cellular utilization and process of energy, stress responses, aging and metabolism, and the immune response, as well as the *SIRT1* enzyme, which utilizes NAD, and in turn, changes in development of the aging process were observed. *SIRT1* also increased longevity of mammals, and the suppression thereof led to cellular senescence. *SIRT1* levels and their protective potential decline with age, resulting in a variety of age-associated diseases 25. Many cancers have been associated with tumor-suppressor miRNAs as down-regulated miR-138, miR-181a, miR-181b, and miR-130b, in addition, miR-137 and miR-668. MiR-130b also targets *p63* mRNA in tandem, noted by several research groups. The *P63* gene is a transcription factor in the *p53* family that plays a pivotal role in epithelium development. Studies *in vivo* and *in vitro* demonstrated that *p63* prevents aging and cellular senescence. Moreover, *p63* directly downregulated the expression of the senescence-related miRNAs, miR-130b, miRNA-138, miRNA-181a, and miR-181b 25. Studies have shown that p63-dependent miRNA regulation of Dicer, as well as TAp63-mediated activation of miR-130b, suppresses tumor growth and metastasis and thereby inhibits tumorigenesis. In keratinocytes, the senescence-associated miRNA miR-191 has been characterized in detail. MiR-191 targets CDK6 and *SATB1*, thereby blocking the G1/S transition, inducing cell-cycle arrest, and promoting a senescent state. In parallel, miR-191 upregulates senescence-associated markers and downregulates CDK6 and *SATB1* expression. *SATB1* is a chromatin organizer that recruits coactivators such as histone acetyltransferases (HATs) and corepressors such as histone deacetylases (HDACs) to gene promoters, thereby acting as a scaffold for chromatin-remodeling complexes 26.

## THE REGULATION OF DIFFERENT MELANOCYTE PROCESSES BY AGE-DEPENDENT MicroRNAs

Melanocytes are specialized epidermal pigment-producing cells that contribute to photoprotection by synthesizing melanin. Their number and function decline with age, contributing to age-related hypopigmentation and hair graying. In human skin, melanocyte density decreases after approximately age 25–30, with reported declines of about 10–20% per decade, although chronically sun-exposed skin may show higher melanocyte density and irregular pigmentation due to altered melanocyte distribution and function 27. Age-dependent miRNAs regulate melanocyte decline by modulating melanogenesis, senescence, proliferation, and survival pathways, particularly through MITF, SIRT1/DNMT1, and p16/Rb signaling 28. In B16-4A5 melanoma cells, miR-141-3p and miR-200a-3p target the MITF 3’UTR, reduce TYR activity, and inhibit melanin synthesis, whereas MITF normally drives expression of key pigmentation genes and supports melanocyte survival and function 29.

These miRNAs exert opposing effects on pigmentation through distinct molecular mechanisms. miR-25 has been reported to suppress MITF, thereby reducing the expression of downstream melanogenic enzymes such as TYR, TYRP1, and DCT and promoting skin lightning. In contrast, miR-21-5p targets SOX5, a transcriptional repressor of MITF, leading to increased MITF and TYR expression and enhanced melanogenesis. Similarly, miR-434-5p promotes skin whitening primarily by directly inhibiting tyrosinase and reducing melanin production 30, 31.

Age-related loss of SCF and α -MSH may exacerbate melanocyte dysfunction and reduce photoprotection. In parallel, miR-34a/c upregulation can promote senescence through SIRT1 suppression and p53/p21 activation, with downstream effects on MITF-dependent melanogenesis 32. Other age-associated miRNAs, including miR-217, have been linked to senescence-associated epigenetic changes through DNMT1 suppression and p16/pRb activation in skin fibroblasts, whereas miR-181a-5p has been reported to inhibit melanogenesis by targeting MITF in a hyperpigmentation model 33, 34.

In melanoma, miR-205 is downregulated and exerts tumor-suppressive effects by targeting *E2F1/E2F5* and inhibiting proliferation, whereas the miR-29 family suppresses melanoma progression in part through repression of MAFG and MYBL2. These findings support the broader concept that tumor-suppressive miRNAs can restrain melanoma growth; however, specific repigmentation strategies such as anti-miR-25 or anti-miR-217 remain insufficiently validated 35–37.

## THE FUNCTION OF MicroRNA REGULATION IN DERMAL FIBROBLAST AGING

The ECM, which is made up of proteins and certain polysaccharides, is abundant in the dermal layer. Most of the dermal cells are fibroblasts. Schwann cells, nerve endings, pericytes, mast cells, tissue macrophages, endothelial cells packed in vessels, and other immune system cells, on the other hand, make up a minority 38, 39. The reticular (lower) and papillary (upper) layers make up the dermis. The reticular dermis has stronger collagen fibers than the papillary layer, which begins at the epidermal basement membrane and is more densely packed with fibroblasts 40. However, post-translational alterations or changes in ECM protein turnover occur with aging, resulting in a loss of ECM structure 41.

Like other cells, fibroblasts produce and replace extracellular matrix components, aid in wound healing, and eventually lose some of their functional abilities 42. There is already a large amount of evidence suggesting that miRNAs can mediate fibroblast functions as well. In senescent skin fibroblasts, miRNAs are linked to a reduction in the production of transmembrane receptors (integrins, for example) and extracellular matrix components (collagens, elastin) 43–45.

In human dermal fibroblasts that are proliferating, miR-152 and miR-181a cause senescence 44. In fact, by specifically targeting and downregulating integrin α 5 (*ITGA5*), miR-152 dramatically reduces cutaneous fibroblast adhesion. Integrin α 5 facilitates cell adhesion and motility as a fibronectin receptor and mediates cell-surface communication. Moreover, *ITGA5* is an essential functional target of miR-205 in the epidermal re-epithelialization process. Downregulation of miR-205 in the migrating epithelial tongue facilitates skin wound re-epithelialization by derepressing *ITGA5*, thereby increasing keratinocyte migration, similar to the effects of TGF- β 1. Notably, elevated miR-181a directly targets and downregulates *collagen XVI (COL16A1)* in senescent fibroblasts. Although *collagen XVI* constitutes only a small fraction of the skin extracellular matrix, it is expressed in fibroblasts, keratinocytes, smooth muscle cells, and amnion. *Collagen XVI* is expressed at the dermal–epidermal junction zone of the papillary dermis, where it mechanically anchors extracellular matrix proteins to cells. Remodeling of the dermal extracellular matrix in aged skin appears to involve both miR-152 and miR-181a in a complex manner 46, 47.

## MicroRNAs AS AGING BIOMARKERS

The expression of various miRNAs in cells, tissues, whole blood, plasma, and serum has been shown to exhibit age-dependent shifts, positioning these molecules as potential systemic biomarkers 48–50. Research assessing the regulation of longevity and age-related pathologies across species has identified key conserved miRNAs—such as let-7, lin-4, miR-17, and miR-34—that are implicated in aging processes and, in some contexts, may contribute to extended lifespans 51–53. Within the specific context of skin physiology (summarized in Table 1), these miRNAs function as critical regulatory hubs for cellular senescence, ECM remodeling, and tissue regeneration. Beyond acting as biomarkers, miRNAs are active drivers of aging-related cellular decline 54. For instance, miR-101 induces human fibroblast senescence through downregulation of Ezh2, and the miR-27a-5p–*SMAD2–MMP1/COL1/BCL2* axis has been identified as a driver of G2/M cell cycle arrest and apoptosis in primary human skin fibroblasts. These regulatory pathways are supported by the essential machinery of miRNA biogenesis, such as Dicer, which is expressed in both the epidermis and the hair follicle’s outer root sheath to ensure consistent miRNA maturation 55, 56. The functional overlap observed in these aging-related miRNAs is further explained by their structural classification; miRNAs are frequently clustered into families based on identical 5 ${}^{\prime }$ seed sequences (nucleotides 2–8), which serve as the primary determinant for mRNA target recognition. This structural categorization allows for a more holistic understanding of skin aging: miRNAs sharing the same seed sequence often exhibit functional redundancy, enabling them to form robust ’regulatory clusters’ that collectively repress essential aging-related pathways. Consequently, the observed aging phenotypes in the skin likely emerge from these coordinated’seed-sharing’ networks rather than the stochastic action of isolated molecules 15, 57, 58.

The roles of different miRNAs in skin physiology are summarized in Table 1.

**Table 1:** The role of different miRNAs in skin physiology .

Type of miRNA	Role in Skin Rejuvenation	Target Genes/Pathways	Applications	References
MIR-21 (Pro-aging)	Inhibition reduces fibroblast senescence and fibrosis	PTEN → PI3K/Akt; Smad7 → TGF- β /SMAD2/3 activation	Anti-fibrotic, wrinkle reduction	59

MIR-29 (Pro-aging)	Inhibition restores collagen synthesis	COL1A1, COL1A2, ECM genes → ↓ matrix production	Dermal thickening, elasticity restoration	60

MIR-146a (Anti-aging)	Suppresses inflammation and oxidative stress	IRAK1, TRAF6 → NF- κ B signaling inhibition	Anti-inflammatory skincare, aging prevention	61

MIR-217 (Pro-aging)	Inhibition reduces fibroblast senescence	DNMT1 → p16/pRb hypomethylation	Anti-senescence therapy	34

MIR-181a (~Pro-aging)	Inhibition reduces mitochondrial stress and senescence	SIRT1, PDIA6, p63, MITF → mitochondrial dysfunction, senescence	Senescence modulation, treatment of hyperpigmentation, restoration of mitochondrial function	62

MIR-126 (Anti-aging)	Enhances angiogenesis and improves blood flow to damaged skin	SPRED1, PIK3R2 → VEGF/PI3K/AKT activation	Skin rejuvenation, vascularization, wound healing	62

MIR-203 (Pro-aging)	Inhibition promotes keratinocyte proliferation and barrier repair	p63, survivin → proliferation vs. differentiation boundary	Skin barrier repair, epidermal renewal, anti-scar	22, 63

MIR-31 (Anti-aging)	Overexpression enhances re-epithelialization and wound closure	EMP1, Rasa1/Spred1 → RAS/MAPK (ERK) → keratinocyte migration	Accelerated wound healing, skin injury repair	64, 65

MIR-135a (Anti-aging)	Overexpression promotes fibroblast migration and ECM production	LATS2 (Hippo pathway) → ↑ fibroblast activation, migration, ECM	Skin elasticity improvement, wrinkle reduction	66

### MIR-377

DNA methyltransferases, of which DNMT1 is the most well-known, regulate DNA methylation. One study demonstrated that young human skin fibroblasts (HSFs) had much higher levels of DNMT1 expression than passage-aged HSFs, and that DNMT1 knockdown considerably accelerated the senescence phenotype in young HSFs. MiR-377 expression is substantially higher in passage-aged HSFs than in immature HSFs. While miR-377 suppression reduced senescence in passage-aged overexpression of miR-377 in HSFs accelerated senescence in young HSFs 8, 34.

To regulate gene expression and other functions, DNMT1 methylates DNA. A study 8 found that the expression of miR-377 was considerably higher in passage-aged HSFs than in young HSFs. Additionally, overexpression of miR-377 in immature HSFs increased the SA- β -gal-positive ratio and *p16* expression while decreasing HSF proliferation. Conversely, inhibition of miR-377 in passage-aged HSFs reduced the SA- β -gal-positive ratio and *p16* expression while increasing cell proliferation.

DNA methylation by DNMT1 is a critical epigenetic process that maintains stable and appropriate gene expression. Dysregulation of DNMT1 has been implicated in numerous illnesses, including autosomal dominant cerebellar ataxia with deafness and narcolepsy (ADCADN), hereditary sensory and autonomic neuropathy type1E (HSAN1E), and several cancers such as colon, gastric, and melanoma 67–69. Elevated miR-377 expression in passage-aged HSFs may accelerate senescence by inhibiting DNMT1 activity.

### MIR-124

Emerging evidence suggests that miR-124 plays a significant role in the molecular mechanisms underlying skin aging. In a comparative analysis of facial skin from young (0–10 years) and elderly (80–100 years) individuals, a miRNA PCR array examining 88 miRNAs involved in human cell differentiation and development revealed a marked overexpression of several miRNAs in senescent skin. Among these, miR-124 exhibited the greatest increase in expression relative to young skin.

To validate these findings, real-time PCR analysis using specific primers for miR-124 was performed on an expanded sample set consisting of six senescent and three young facial skin samples. The results confirmed a significant elevation of miR-124 levels in senescent skin, supporting its involvement in age-related molecular alterations of the epidermis. These data indicate that miR-124 may contribute to the regulation of cellular senescence pathways in human skin, potentially influencing processes such as keratinocyte differentiation, proliferation, and stress response 70.

## THE MIR-34 FAMILY

The miR-34 family (miR-34a, miR-34b, miR-34c) is directly induced by *p53* and is one of the best-characterized miRNA groups in aging and cellular senescence. In human skin, miR-34a is upregulated in both chronologically aged and photoaged samples, including higher expression in sun-exposed compared with sun-protected sites across different age groups, which indicates that its expression reflects a combination of intrinsic aging and cumulative UV damage. Members of the miR-34 family promote senescence by causing G1 cell-cycle arrest and suppressing proliferation, acting through inhibition of key targets such as cyclin D1, CDK4, SIRT1, and other pro-survival or pro-regenerative factors. In primary keratinocytes and embryonic skin, miR-34-mediated repression of these targets leads to a senescent-like phenotype, reduced regenerative capacity, and enhanced apoptosis. Beyond its direct effects on cell-cycle control, miR-34 also contributes to the structural manifestations of skin aging. In the dermis, increased miR-34 activity is linked to activation of matrix metalloproteinases (MMPs) and subsequent collagen degradation, thereby promoting extracellular matrix deterioration and wrinkle formation. Pathway analyses of miR-34a targets in aged or photo exposed skin highlight enrichment in adherens junction, MAPK, Notch, and p53 signaling pathways, underscoring its central role at the intersection of DNA damage responses, stress signaling, and tissue remodeling. Taken together, these findings support the view that the miR-34 family functions as a key pro-aging miRNA cluster in skin, acting upstream as a p53-responsive driver of keratinocyte and fibroblast senescence and downstream as a facilitator of collagen breakdown and photoaging-associated structural changes 71–75.

## THE MIR-39 FAMILY

The miR-29 family (miR-29a, miR-29b, miR-29c) is best known for its role as a central negative regulator of ECM genes, including multiple collagens, fibronectin, laminin, and other matrix-associated proteins. Numerous experimental studies in fibroblasts and tissues have shown that miR-29 directly targets mRNAs encoding fibrillar collagens such as *COL1A1, COL1A2, COL3A1*, as well as basement-membrane components like type *IV collagen*, leading to reduced collagen synthesis and altered matrix organization. In the context of aging, miR-29 expression is consistently found to be elevated in several organs and models, and this upregulation correlates with downregulation of collagen and ECM pathways and with phenotypes such as tissue thinning, weakened structural support, and impaired repair capacity. Recent *in vivo* work has strengthened the view of miR-29 as an active driver of aging-related changes rather than a passive marker. In mouse models, overexpression of miR-29 is sufficient to induce multiple features of an aging-like phenotype, including reduced skin thickness and abnormal collagen deposition in the dermis and epidermis, while genetic or experimental reduction of miR-29 can delay certain premature aging manifestations and extend lifespan in accelerated aging (progeria-like) settings. These findings indicate that excessive miR-29 activity promotes an imbalance between collagen synthesis and degradation that contributes to structural frailty of tissues, including skin. For cutaneous aging, the miR-29 family can therefore be framed as a pivotal anti-fibrotic miRNA cluster: its age-associated induction suppresses collagen production and basal-membrane integrity, cooperates with matrix-degrading enzymes to drive dermal thinning and wrinkling, and represents a candidate target for interventions aimed at preserving or restoring dermal ECM in aged skin 60, 76–78.

### MIR-302b3p

miR-203b-3p has recently been identified as a senescence-associated miRNA with a direct impact on epidermal stem-cell maintenance and structural aging of the epidermis. Increased expression of miR-203b-3p has been observed in aged compared with young skin in both human and murine models, suggesting that its upregulation is a conserved feature of cutaneous aging. Functionally, miR-203b-3p targets the mRNA of *collagen type XVII* α *1 (COL17A1)*, a key hemidesmosomal component that anchors basal keratinocytes to the basement membrane and is essential for epidermal stem-cell adhesion, polarity, and long-term fitness. By binding to the 3 ${}^{\prime }$ -untranslated region of *COL17A1*, miR-203b-3p suppresses its expression, leading to reduced *COL17A1* protein levels and compromised hemidesmosome integrity in aged epidermis.

Experimental models have shown that loss or downregulation of *COL17A1* results in diminished clonogenic capacity of epidermal stem cells, thinning of the epidermal layer, and increased skin fragility, all of which are characteristic features of aging skin. The age-dependent rise in miR-203b-3p therefore likely contributes to epidermal atrophy and impaired regenerative potential by weakening the structural and molecular niche required for stem-cell maintenance. In this context, miR-203b-3p can be viewed as a pro-aging miRNA that links altered post-transcriptional regulation to stem-cell decline and barrier deterioration in aging skin. This mechanistic axis highlights miR-203b-3p and *COL17A1* as a potential target pair for interventions aimed at preserving epidermal stem-cell function and improving the structural integrity of aged skin 79–81.

### MIR-361-5p

MiRNA patterns linked to cellular senescence and polyphenol-induced senescence reversal were found in a study conducted in human primary endothelial cells. They discovered that in response to senescence and rejuvenation, miR-361-5p expressed itself differentially. The study found that applying miR-361-5p mimic to late passage human endothelial cells reduced the culture’s senescent burden by 14%. RNAi gene knockdown of conserved miR-361-5p target genes, however, resulted in adverse effects on longevity and/or health span in a *C. elegans* model. Even though miR-361-5p may reduce some aspects of the senescence phenotype in human primary endothelial cells, several of its validated target genes are necessary for the formation or regulation of the cytoskeletal network or its interface with the extracellular matrix. These processes are essential for both cell survival and cell function 82.

### MIR-146a

Since miR-146a interacts with several biological processes associated with skin aging, including collagen synthesis, cell-to-cell communication, cell damage repair, cell proliferation, and circadian rhythm machinery, it might be an aim for anti-aging skin care. *Adansonia digitata* extract can boost miR-146a expression, which benefits skin cell health. Skin fibroblasts were treated with an extract from the *Baobab tree* (*Adansonia digitata*) to prevent the negative effects of miR-146a downregulation. Numerous biological characteristics of baobab, including its antiviral, antioxidant, and anti-inflammatory qualities, may aid in reversing the aging process of the skin and promoting skin rejuvenation. The expression levels of miR-146a were measured in normal human dermal fibroblasts (NHDFs) from donors who were 19, 27, 40, and 62 years of age. While the hsa-mirVana miR-146a inhibitor decreased miR-146a expression, the *Adansonia digitata* extract increased it. The Impacts on skin aging indicators such as collagen-1 synthesis, cell proliferation, and inflammatory cytokines were evaluated. They demonstrate that dermal fibroblasts’ expression of miR-146a declines with age, and that blocking miR-146a in NHDFs between the ages of 19 and 62 led to notable alterations in key clock genes, suggesting a connection to circadian rhythm regulation. Moreover, DNA damage, collagen-1 synthesis, cellular proliferation, and pro-inflammatory indicators are all decreased by downregulating miR-146a. When miR-146a was activated with the *Adansonia digitata* extract, the negative effects of miR-146a inhibition were lessened, and miR-146a transport by exosome transfer increased 83.

### MIR-21

The development of pathological skin disorders, such as psoriasis, cancer, and poor wound healing, is significantly influenced by miRNA-21 84 . A study by 85 looked at the expression of miR-21 in the skin of human female donors (ages 48, 60–62, and 78) and mice of different ages (8 weeks vs. 2 years). As a control for this study, the amount of miRNA-199a, which is not present in the epidermis of either human or mouse skin, stayed constant as human skin aged, in contrast to miR-21. Pro-miR-21 mimic or *SATB1*-expressing lentiviral particles were used to overexpress *SATB1* and miR-21 in keratinocytes to investigate the potential functional link between miR-21 and *SATB1* in skin aging. Using RT-qPCR, we verified that primary mouse epidermal keratinocytes exhibited increased levels of *Satb1* (*SATB1* Leti + miR-Control) or miR-21 (Control Leti + pro-miR-21). They show that *SATB1* expression and miR-21 have an inverse relationship in both young and old skin, that miR-21 is indeed directly targeted by *Satb1* in keratinocytes, and that miR-21 expression is higher in aging skin in both humans and mice. Consequently, by controlling *SATB1* in epidermal keratinocytes, miR-21 may make people more vulnerable to age-related degenerative disorders like cancer. Additionally, it might foster the development of enhancer-promoter networks and higher-order chromatin remodeling, both of which are critical for epidermal differentiation 85.

### MIR-181a

During replicative senescence, keratinocytes have up-regulated levels of miR-181a, which target *p63* and *Sirtuin 1* mRNAs and affect cell proliferation pathways. This up-regulation is connected to the aging process in skin cells and results in aging symptoms and a decline in cellular function. In dermal fibroblasts, miR-181a-5p is one of the aging indicators that influence telomere preservation and various phases of the cellular life cycle. This suggests that miR-181a plays a role in the broader context of cellular aging as well as in keratinocytes. MiR-181a has been implicated in skin immunosenescence, specifically affecting Langerhans cells. A diminished immune response may result from the failure of these cells, which are essential for the skin’s immunological response, because of miR-181a upregulation in aging skin 86. Human amniotic stem cell (hASC) exosomes produce miR-181a-5p, which has been demonstrated to suppress melanogenesis by reducing MITF (microphthalmia-associated transcription factor), a crucial regulator of melanin formation. Hyperpigmentation, a typical problem with aging skin, is lessened by this technique. Furthermore, by triggering autophagy, miR-181a-5p encourages the breakdown of melanosomes. MiR-181a may be used to treat age-related pigmentation problems because of its dual roles in preventing melanin synthesis and encouraging its breakdown 87.

### MIR-155

miR-155 has emerged as a key regulator that affects keratinocyte (KC) development and has significant therapeutic potential in the context of skin rejuvenation. MiR-155’s function has been investigated in clinical trials, *in vitro*, and *in vivo*. *In vitro*, it has been demonstrated that miR-155 negatively regulates keratinocyte differentiation. In human skin models, it has been demonstrated that inducing miR-155 to overexpress results in a marked diminished expression of Loricrin, a protein essential for forming the skin’s protective barrier. While miR-155 expression is downregulated during keratinocyte differentiation in healthy skin, it is significantly upregulated in psoriatic lesions, indicating that miR-155 contributes to disrupted keratinocyte differentiation in skin disorders 88 . The function of miR-155 in the inflammatory milieu of psoriatic skin has been highlighted by the demonstration that pro-inflammatory cytokines such as IL-17, IL-1 β , and IFN- γ cooperate to increase miR-155 expression in keratinocytes during inflammatory conditions. Potential therapeutic prospects for skin rejuvenation by miR-155 modulation are suggested by the complex link between miR-155 and keratinocyte activity 89.

Further evidence for the role of miR-155 in skin health and rejuvenation comes from *in vivo* studies. Studies using mouse models of skin diseases, such as atopic dermatitis (AD), have shown that silencing miR-155 can decrease epidermal thickness and inflammatory cell infiltration, restoring the function of the skin barrier 90. Modifying tight junction (TJ) proteins, which are essential for maintaining the integrity of the epidermis, is also associated with targeting PKI α , a direct target of miR-155-5p. TJ protein repair after miR-155-5p suppression implies that it is essential for maintaining the skin’s barrier function in inflammatory conditions 91. Another study showed that by inhibiting the JAK/STAT3 signaling pathway, miR-155 knockdown decreased inflammation and autoimmune disease in rats with chronic skin diseases 92 . This implies that by addressing the underlying inflammatory processes in chronic skin illnesses, targeting miR-155 may improve skin regeneration. The development of miRNA-based therapeutics for dermatological conditions is of great interest, despite the paucity of clinical trials investigating miR-155 alteration for human skin rejuvenation currently. The field of miRNA therapeutics has shown promise overall, despite the lack of information regarding clinical trials that specifically target miR-155. Research focuses on developing small chemicals and delivery systems that effectively alter miRNA expression 93. As our knowledge of miR-155 to skin illnesses continues to expand, future clinical trials are anticipated to explore its modulation as a potential therapeutic technique for skin renewal and repair.

### MIR-22

It has been discovered that miR-22 is an essential miRNA that plays a significant role in skin renewal, especially when taking keratinocyte function and skin health into account. In addition to therapeutic application implications, MiR-22 research includes both *in vitro* and *in vivo* investigations. In keratinocytes, miR-22 is known to control both cell division and apoptosis. By triggering the Wnt/ β -catenin signaling pathway, which is essential for keratinocyte maintenance and differentiation, it enhances stem cell characteristics 94.

It has been demonstrated that miR-22 alters pro-inflammatory reactions in cutaneous models. It may play a function in skin rejuvenation treatments that target inflammation because it takes part in inflammatory processes that are frequently dysregulated in skin disorders 95.

Research has shown that miR-22 plays a vital role in skin development, influencing skin regeneration by regulating hair follicle regression and aiding in the differentiation of hair follicle stem cells (HFSCs) 96. By altering cellular behavior and encouraging apoptosis in sick cells, miR-22 may protect against diseases like cutaneous T-cell lymphoma, according to research on skin disorders 97. This implies that increasing miR-22 activity may help the skin in diseased conditions heal and regenerate. Although there aren’t many clinical trials focusing on miR-22 for skin renewal, there is the promise of creating treatments based on its modulation. Future research may concentrate on miR-22’s therapeutic uses in clinical settings, namely targeting skin aging and illnesses, given its documented functions in cellular differentiation and inflammatory responses 98.

### MIR-23a

Mir-23a-3p, the major mature strand derived from the MIR-23A precursor, has been identified as a pro-senescence miRNA in skin. Its expression is increased in aged human dermal fibroblasts and in aged murine skin, where it directly targets hyaluronan synthase 2 (HAS2), leading to downregulation of HAS2 and a consequent reduction in hyaluronic acid (HA) synthesis. This loss of HA, a key determinant of dermal hydration, viscoelasticity, and extracellular matrix organization, is accompanied by an upregulation of senescence-associated markers, including increased SA- β -gal activity and p21, and a reduction in fibroblast proliferative capacity. Overexpression of miR-23a-3p in young fibroblasts reproduces this senescent phenotype, whereas pharmacological inhibition of HA synthesis or knockdown of HAS2 mimics the effects of miR-23a-3p, confirming that the miR-23a-3p–HAS2–HA axis is functionally implicated in fibroblast aging 99.

### MIR-24

The significance of miR-24 for skin rejuvenation is becoming more widely recognized, especially in keratinocyte differentiation, proliferation, wound healing, and inflammation response. Numerous facets of skin health that are essential for rejuvenation treatments have been the focus of research investigations looking into miR-24. According to research, miR-24 triggers epidermal differentiation via controlling actin cytoskeleton remodeling, which is essential for the skin’s structural integrity and functionality. Keratinocyte migration and adhesion, two crucial processes in skin repair and renewal, are impacted by this mechanism. MiR-24 has been shown to affect the growth and multiplication of primary keratinocytes. Its expression is specifically increased during keratinocyte differentiation, suggesting that it may help transition from a proliferative to a differentiated state. According to studies, miR-24 influences the migration and proliferation of skin fibroblasts, both of which are necessary for efficient wound healing. Its control could affect the regeneration of skin after injury, improving skin rejuvenation outcomes. MiR-24 has been connected to the control of collagen synthesis and other extracellular matrix components, which are crucial for maintaining the skin’s elasticity and structure. Rejuvenating treatment techniques may target miR-24 since variations in collagen levels can directly affect the resilience and texture of the skin 100.

### MIR-125b

miR-125b has emerged as a key miRNA in skin rejuvenation research, particularly about keratinocyte differentiation, proliferation, and the inflammatory response associated with skin conditions. The following paper summarizes key findings from many studies on miR-125b in connection to skin regeneration. Research has shown that miR-125b is downregulated in conditions like psoriasis, indicating that it controls keratinocyte differentiation and proliferation. This suggests that targeting miR-125b may enhance keratinocyte activity, which is essential for the integrity and regeneration of the epidermal barrier. One of the signaling pathways that miR-125b is involved in is the Fibroblast Growth Factor Receptor 2 (FGFR2) pathway, which is crucial for keratinocyte proliferation and differentiation. MiR-125b may be a helpful therapeutic target for improving skin health because of its capacity to modulate various pathways 101.

Recent studies have shown that increasing miR-125b expression significantly expedited wound healing in elderly mice. This improvement was attributed to increased myofibroblast differentiation and fibroblast migration, both of which are required for efficient wound healing. The study found that exosomes carrying miR-125b are released by young fibroblasts, which promote the migration and transformation of old fibroblasts into myofibroblasts, ultimately promoting wound healing 102. ECM deposition is facilitated by miR-125b through the fibroblast-to-myofibroblast transition (FMT), which is essential for wound healing. It has been shown that its overexpression in exosomes generated from young fibroblasts restores the ability of aged fibroblasts to regenerate.

miR-125b controls the expressiveness of the key inflammatory genes, including those that are part of the NF- κ B signaling cascade. This modulation is especially crucial in inflammatory skin conditions like psoriasis, where an imbalance in inflammatory responses can make skin problems worse 103.

### MIR-7 and let7

MiRNA-7 and the let-7 family both participate in pathways relevant to skin biology, but experimental evidence places them at different levels of validation in the context of skin aging. miR-7 is primarily supported by *in vitro* and preclinical studies, whereas let-7 has been integrated into broader experimental frameworks on skin and organismal aging 104, 105.

In keratinocyte models, modulation of miR-7 has been shown to alter proliferation and differentiation marker expression, indicating a role in epidermal renewal and barrier maintenance, and experimental manipulation of miR-7 levels can reduce pro-inflammatory cytokine production in skin cells, supporting an anti-inflammatory, potentially “pro-youthful” effect. In dermal-oriented systems, overexpression or inhibition of miR-7 has been linked to changes in collagen synthesis and degradation, partly via effects on matrix metalloproteinases, and some UV/oxidative stress models report that miR-7 upregulation increases antioxidant enzyme expression and improves cellular resistance to reactive oxygen species. These findings collectively suggest that miR-7 can enhance regenerative capacity and stress resilience, but they are largely confined to cell culture and experimental models, with limited direct demonstration in aged human skin 106.

By contrast, let-7 family members have been repeatedly implicated in experimental studies of aging. In primary human dermal fibroblasts, age-associated increases in let-7 expression correlate with reduced proliferative capacity and altered extracellular matrix gene expression, and experimental overexpression of specific let-7 isoforms can suppress pro-regenerative or pro-collagen transcripts, consistent with a more “senescent-like” phenotype. In animal and tissue models, modulation of let-7 impacts pathways such as cell cycle control, stem/progenitor cell function, and chronic inflammatory signaling, all of which are central to organismal and cutaneous aging. UV-exposure and stress-response experiments further show that let-7 is part of the miRNA signature that shifts in response to cumulative damage, aligning its expression profile with established aging and photoaging markers. Taken together, experimental data support presenting let-7 as a well-validated miRNA axis directly involved in fibroblast aging, matrix remodeling, and stress-driven skin changes, while miR-7 is more appropriately described as an emerging regulator with promising but still predominantly preclinical evidence for roles in regeneration, inflammation control, and protection against environmental insults 107.

### MIR-223

Research indicates that miR-223 inhibits apoptosis and promotes skin cell proliferation, particularly in keratinocytes that have been stimulated by IL-22. This occurs through the PTEN/Akt signaling pathway, highlighting miR-223’s role as a promoter of skin cell proliferation 108. Numerous studies indicate that miR-223 promotes the production of collagen, which is necessary to maintain the skin’s suppleness and integrity. For instance, it has been shown that miR-223 mimics increase the secretion of alpha-smooth muscle actin ( α -SMA) and collagen I and III in cultured cardiac fibroblasts, suggesting that skin fibroblasts may experience a similar effect 109. This finding demonstrates how miR-223 might promote the production of extracellular matrix components that support skin repair and regeneration.

Research indicates that miR-223 might be a suitable target for skin-rejuvenating therapies. Because miR-223 promotes collagen synthesis, cell proliferation, and inflammation reduction, its usage in treatment regimens may enhance skin health and rejuvenation 110.

### MIR-130b

MIR-130b has been implicated as an important regulator of epidermal and dermal homeostasis. In keratinocytes, miR-130b-3p targets *desmoglein 1 (Dsg1)*, a key component of desmosomes that is required for proper keratinocyte growth and stratification; functional studies have shown that miR-130b-3p inhibits *Dsg1* expression and thereby restrains keratinocyte proliferation and differentiation. These findings suggest that modulation of miR-130b levels could influence epidermal regeneration by fine-tuning *Dsg1-*dependent keratinocyte dynamics. In line with this, experimental overexpression of miR-130b in keratinocytes effectively suppresses differentiation marker expression but does not, by itself, markedly accelerate senescence, indicating that miR-130b primarily maintains a more progenitor-like, proliferative state rather than directly driving senescence. Under pro-senescence stressors such as replicative exhaustion, oxidative stress, or UV exposure, upregulated miR-130b promotes keratinocyte progenitor proliferation by suppressing differentiation (via Δ Np63/Dsg1 targeting), potentially delaying terminal differentiation while contributing to senescence-like phenotypes in the epidermal compartment 111.

In the dermis, miR-130b has been linked to the control of collagen synthesis and fibroblast activation, processes that are critical for maintaining skin elasticity and structural integrity. In fibrotic and scarring models, elevated miR-130b expression correlates with enhanced fibrotic responses, including increased collagen deposition and myofibroblast activation, suggesting that miR-130b promotes a pro-fibrotic phenotype. This dual involvement in epidermal regulation and dermal fibrosis has spurred interest in miR-130b as a therapeutic target. Modulating miR-130b through miRNA mimics or inhibitors may, in principle, be exploited either to support regenerative responses (for example, by transiently enhancing keratinocyte proliferative potential) or to attenuate pathological fibrosis and thus holds potential relevance for both skin rejuvenation and anti-scarring strategies 112.

### MIR-203

It is well established that keratinocyte development depends on miR-203, the predominant keratinocyte-specific miRNA in the epidermis 113. Research indicates that miR-203 has an antiproliferative effect that promotes keratinocyte differentiation and inhibits excessive proliferation. It specifically targets transcription factors such as their 3’ untranslated region (UTR) and p63, which is an essential regulator of keratinocyte stemness and proliferation. Additionally, miR-203 possesses anti-inflammatory properties, which are critical for skin health and rejuvenation. By focusing on pathways linked to inflammatory reactions, miR-203 may reduce chronic inflammation, which contributes to skin aging, according to research 114. Its ability to lessen inflammation-related skin issues has been confirmed by the discovery that keratinocytes with overexpressed miR-203 release less inflammatory cytokines 115 . Collagen is necessary to maintain the skin’s suppleness and structure, and miR-203 has been connected to regulating collagen synthesis. It has been demonstrated that anti-miR-203 treatments influence extracellular matrix remodeling by enhancing collagen reassignment and wound closure 116. Therefore, modifying miR-203 levels could be a therapeutic strategy to promote the skin’s flexibility and structural integrity. MiR-203 may be useful in treating dermatological issues like psoriasis and chronic wounds due to its regulatory functions. Targeting key genes involved in skin regeneration, it offers a path toward developing treatments that enhance wound healing and cosmetic skin rejuvenation 117. Researchers are increasingly looking into MiR-203 as a possible target for therapeutic approaches that encourage skin rejuvenation and repair. Figure 2.

**Figure 2:**
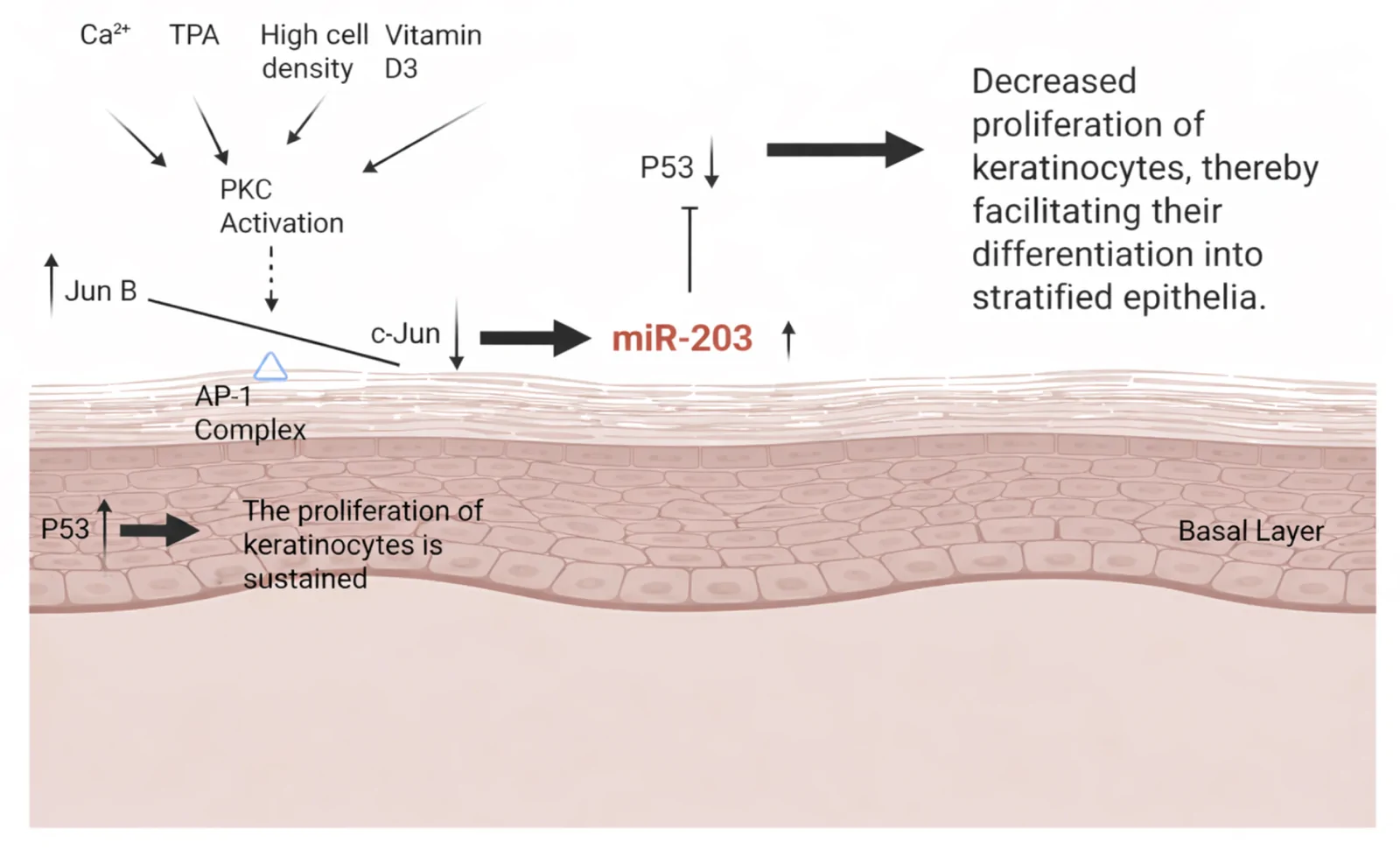
miRNA-203 functions as a regulatory switch that balances keratinocyte proliferation and differentiation in the adult epidermis, thereby playing a key role in skin morphogenesis. Key related factors include TPA (12-O-tetradecanoylphorbol-13-acetate), which activates PKC (protein kinase C), and AP-1 (activator protein-1), which are involved in this regulatory process.

### MIR-217

miR-217 has emerged as a key pro-aging miRNA that accelerates human skin fibroblast senescence through epigenetic reprogramming of classical senescence pathways. In aged human skin tissues and serially passaged dermal fibroblasts from elderly donors, miR-217 expression is markedly upregulated and exhibits a strong inverse correlation with DNMT1 levels 34. Functional studies demonstrate that miR-217 directly targets the 3 ${}^{\prime }$ untranslated region of DNMT1 mRNA, suppressing its translation and thereby impairing maintenance DNA methylation capacity. This epigenetic disruption leads to promoter hypomethylation and derepression of the canonical senescence effectors p16^INK4a and phosphorylated retinoblastoma protein (pRb). The resulting molecular cascade manifests as increased senescence-associated β -galactosidase (SA- β -gal) activity, elevated *p16* and phospho-Rb protein levels, and significantly reduced proliferative capacity in fibroblasts. Gain-of-function experiments confirm causality: overexpression of miR-217 in young, proliferative human skin fibroblasts induces a full senescence program, while inhibition of miR-217 in passage-aged fibroblasts partially reverses the senescent phenotype, restoring proliferation and reducing SA- β -gal positivity 118, 119. Notably, re-expression of DNMT1 in miR-217-overexpressing cells rescues the senescence phenotype, establishing the miR-217 → DNMT1 → p16/pRb axis as the primary mechanism. These findings position miR-217 as a mechanistically defined driver of dermal fibroblast exhaustion, contributing to the age-related decline in extracellular matrix production and dermal structural integrity. Therapeutic inhibition of miR-217 therefore represents a promising strategy for mitigating fibroblast senescence and preserving skin regenerative capacity, highlighting the potential of epigenetic miRNA modulation in anti-aging dermatology 120.

## CONCLUSION

One emerging area of investigation has been to elucidate these involved molecular machineries of skin health associated with aging through the mediated mechanisms of miRNAs and their action in skin repair. This miRNA-mediated regulation of major aspects of bio-physiological action such as keratinocyte proliferation, inflammation, differentiation and extracellular matrix remodeling is facilitated especially miR-203, miR-130b, miR-223, and miR-21. Several miRNAs have been shown to regulate the expression level of keratinocyte-linked genes. In other words, miR-203 was found to maintain an efficient epidermal barrier through differentiation and regulate excessive proliferation to name a few. In like manner, miR-223 enhances keratinocyte migration and re-epithelialization in wound healing, highlighting its powerful therapeutic potential in regenerative dermatology. The let-7 family acts to modulate the abnormal cellular functions commonly observed in skin aging and disease by inhibiting carcinogenic pathways. Moreover, there is evidence to support the anti-inflammatory role of miRNAs in skin rejuvenation. The ongoing lifelong inflammation effects that cause skin diseases and contribute to premature aging can be alleviated by the modulation of inflammatory cytokines expression, and therefore miRNA-modulated inflammatory pathways are important to personalize inflammatory disease therapies and provide a strategic path forward. Since miRNAs affect collagen synthesis and the extracellular matrix structure, this demonstrates that they possess medicinal potential, as they are important for dermal structural and/or functional health and well-being. This paves the way for new therapeutic approaches that could find miRNAs to inhibit aging markers of skin, reactivate, even restore skin health, and enhance clinical outcome in various skin diseases. The application of miRNA in clinical practice can change the paradigm of skin rejuvenation. To clarify the whole potential of miRNAs as therapies, more work has been conducted on miRNA-specific functions, as well as specific regulatory networks. The research on miRNA-based treatments is also underdeveloped, so there are areas where researchers have opportunities for fresh and innovative solutions to tackle issues in aging that go beyond mere facials. This also includes skin health maintenance and benefits.

## CONFLICT OF INTEREST

The authors declare no conflict of interest.
